# MicroRNAs: emerging biomarkers and therapeutic targets in pancreatic cancer

**DOI:** 10.3389/fmolb.2024.1457875

**Published:** 2024-09-03

**Authors:** Jiaqian Yuan, Kaiqi Yan, Yong Guo, Yan Li

**Affiliations:** ^1^ The First Clinical Medical College, Zhejiang Chinese Medical University, Hangzhou, China; ^2^ Department of Materials Engineering and Science, Ningbo University of Technology, Ningbo, China; ^3^ Department of Medical Oncology, The First Affiliated Hospital of Zhejiang Chinese Medical University, Hangzhou, China

**Keywords:** microRNA, pancreatic cancer, biomarker, treatment, prognosis

## Abstract

Pancreatic cancer (PC) is a highly malignant disease with high aggressiveness and a dismal prognosis, which is challenging to diagnose clinically early and gains low benefit from standard therapies. MicroRNAs (miRNAs) have become a hot topic in oncology research. Current evidence indicates that miRNAs are regulators involved in the entire process of PC, providing new diagnostic and therapeutic strategies for this fatal disease. Related research has been rapidly updated, making it necessary to review it to propose new directions and ideas and provide guidance for the development of precision medicine for PC. We reviewed the relevant literature through Pubmed, Embase, Web of Science and Medline, showing that abnormally expressed miRNAs in PC patients have the potential to be used as biomarkers for diagnosis and prognosis, highlighting the excellent prospect of combining miRNAs with traditional therapies, and the effective application of these factors for PC, especially miRNA mimics and inhibitors. MiRNAs participate in the entire process of PC and play important roles in diagnosis, treatment and prognosis. They are potential factors in conquering PC in the future.

## 1 Introduction

Pancreatic cancer (PC) is a highly invasive malignant tumor and the seventh most lethal cancer category worldwide (Rawla et al., 2019). According to a study (Amini et al., 2023), the global mortality and incidence of PC have remained at an annual increase of 0.8% in the past 30 years, posing a serious threat to human health and life. The most common pathologic type of PC is pancreatic ductal adenocarcinoma (PDAC), which accounts for more than 90% (Vincent et al., 2011). This malignant disease has no characteristic early clinical manifestations; its imaging features are insidious, making early diagnosis difficult. Its rapid progression and poor prognosis make it very challenging clinically. Currently, therapies for PC are mainly surgery, perioperative and advanced radiotherapy, chemotherapy, and immunotherapy. However, only 15%–20% of PC patients have the opportunity to undergo surgical treatment (Halbrook et al., 2023). Moreover, the disease has poor sensitivity to these conventional therapies, which may be due to the dense connective tissue hyperplasia of PC tissue and the highly immunosuppressive tumor microenvironment (TME), which prevents related chemotherapy drugs and small molecule inhibitors from exerting anti-cancer effects. Despite the effective use of these medications, they are prone to developing resistance soon after (Sherman and Beatty, 2023).

**FIGURE 1:**
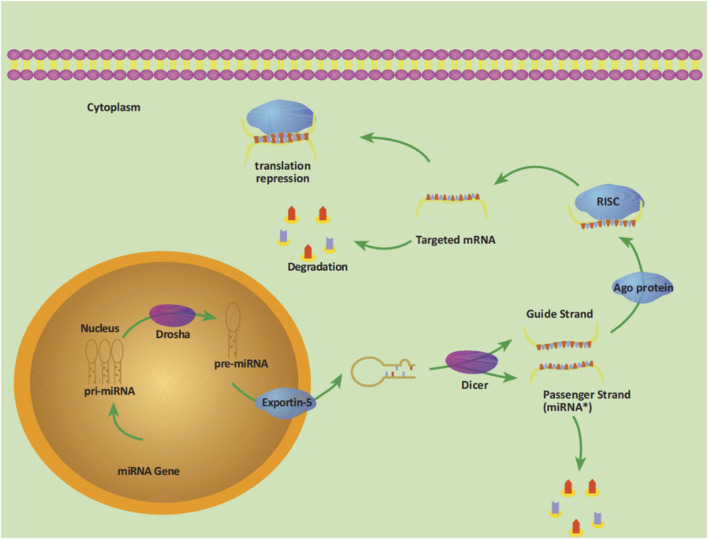
The process of miRNA production. The pri-miRNA (primary transcript) with a length of about 300–1,000 bases transcribed by the miRNA gene in the nucleus is cleaved by the RNase III-Drosha enzyme into a hairpin structure precursor miRNA (pre-miRNA), with a length of about 70–90 bases. Pre-miRNA is transported into the cytoplasm under the action of the transporter exportin-5 and then further cleaved by another RNase III-Dicer enzyme. One of the double strands is the Passenger Strand (miRNA*), which is eventually degraded, and the other strand is the Guide Strand, together with the Argonaute protein (Ago) to form RISC, which promotes the binding of miRNA to targeted mRNA, thereby degrading target mRNA or inhibiting translation.

MicroRNAs (miRNAs) are a class of non-coding single-stranded RNA molecules encoded by endogenous genes with a length of approximately 19–24 nucleotides, usually located in intergenic or intronic regions, which are highly conserved in evolution and have the function of regulating posttranscriptional gene expression and protein expression. The production of miRNA begins in the nucleus. The pre-miRNA is transported to the cytoplasm and cleaved by Dicer enzymes to produce mature miRNA, which forms an RISC (RNA-induced silencing complex) with other proteins, binding to the 3′-untranslated region (3′-UTR) of the target mRNA, thereby degrading the target mRNA or inhibiting translation (He et al., 2020). The entire process is shown in Figure 1. Each miRNA can regulate multiple target genes, and a specific targeted mRNA can also be regulated by multiple miRNAs at the same time. Dysregulation of any miRNA can potentially disrupt signaling pathways through their targets, facilitating the development or progression of cancer. The expression profile of miRNAs shows that miRNA expression is differentially expressed in tumor tissue compared with normal tissue and is specific in different malignancies (Daoud et al., 2019). Given the highly aggressive, difficult-to-treat, and poor prognosis of PC, it is necessary to explore new therapeutic breakthroughs. The field of miRNA research has been rapidly developing and updated. Based on recent literature, we conducted relevant summaries and analyses to explore the role of miRNAs in the pathogenesis, diagnosis, treatment, and prognosis of PC. Furthermore, the study also aims to provide novel viewpoints on managing this highly malignant disease in the clinic and new directions for more in-depth basic and clinical research.

## 2 The role of miRNAs in pathogenesis

PC is a complex disease caused by the interaction of genetic and environmental factors. A study revealed that (Wood et al., 2022) the occurrence of PC is accompanied by a large number of gene mutations, with the main driving genes include *KRAS*, *TP53*, *SMAD4*, and *CDKN2A*. In addition, abnormal epigenetic modifications also promote the occurrence and development of PC by regulating chromatin structure and gene expression. MiRNAs, as a post-transcriptional regulator, have long been shown to be closely related to the pathogenesis of PC (Roldo et al., 2006). Studies have demonstrated (Hanoun et al., 2010; Wang P. et al., 2014) that both miR-148a and miR-124 are downregulated during the oncogenic process of PDAC due to epigenetic regulation, specifically DNA methylation. MiRNAs are not only involved in the epigenetic regulation of PC development, but also act as regulatory factors for epithelial-mesenchymal transition (EMT).Gurbuz et al. (2022) discovered that downregulated miR-193b can prevent the proliferation, migration, invasion, and EMT of PC cells by inhibiting the eEF2K/MAPK-ERK axis. They additionally found that the expression of miR-193b in PDAC cells is significantly insufficient compared with that in normal pancreatic epithelial cells (Gurbuz et al., 2022). MiR-33a-3p has also been shown to regulate AREG (amphoteric regulatory protein) mediated by METTL3 (methyltransferase-like 3), thereby altering EMT and inhibiting PC invasion and metastasis (Su et al., 2023).

Upregulation of multiple oncogenic miRNAs (oncomiRs) or silencing of tumor suppressor miRNAs (tsmiRs) enhances the activity of targeted oncogenes, thereby causing cancer. MiR-21 has been demonstrated to be overexpressed in early PC and PC-derived cell lines, which are generally considered downstream of the carcinogenic pathway, regulating targeted genes through pathways such as *KRAS* (Du Rieu et al., 2010; Moriyama et al., 2009). Relevant research on the role of miR-21 in PC is ongoing. In recent years, Zhao et al. (2018) have proven that miR-21 targets Spry2 (Sprouty2) and regulates cell proliferation through MAPK/ERK and PI3K/AKT signaling pathways to promote EGF-induced (epidermal growth factor) proliferation of PC cells. Conversely, the downregulation of miR-21 leads to elevated expression of the tumor suppressor *VHL*, resulting in the inhibition of proliferation, migration, and invasion of PC cells (Sun et al., 2019). In clinical samples of PC, miR-222 has been found to be upregulated with increased invasiveness, which is caused by targeting p57, ultimately leading to increased viability of PC cells *in vitro* (Zhao et al., 2015). Nevertheless, the upregulation of certain miRNAs inhibits tumor development. Overexpression of miR-503-5p substantially decreased cell viability, induced apoptosis, caused G0/G1 arrest, and inhibited cell migration (Li et al., 2024). In a study conducted by Liang et al. (2023), it was conclusively demonstrated that the expression of miR-23a in PC cells leads to a downregulation of FOXM1 (Forkhead box M1) expression, which inhibits the proliferation and migration of PC cells. Importantly, miR-23a and FOXM1 were identified as mutually negative regulatory factors in the expression context of PC cells, suggesting a complex interplay between these two molecules in modulating the malignant disease. These findings provide valuable insights into the molecular mechanisms underlying PC progression and may offer potential therapeutic targets for the management of this aggressive malignancy.

In addition, blood vessels in tumor tissue exhibit the biological characteristics of low reactivity, high permeability, and low oxygen supply capacity, and the progression of PC is positively correlated with blood vascular density. Yang et al. (2021) verified that miR-155-5p and miR-221-5p entered endothelial cells by targeting *E2F2* (E2F transcription factor 2) to induce angiogenesis and growth of PDAC based on *in vitro* and *in vivo* experiments. In contrast, miR-29b reduces the angiogenesis of PC via ROBO1 (roundabout guidance receptor 1) and SRGAP2 (SLIT-ROBO Rho GTPase activating protein 2) (Wang et al., 2022). MiR-145 functions as a tumor suppressor in PC cells by targeting angiopoietin-2 (Ang2) to inhibit translation, thereby suppressing the invasion and growth of PC cells. It has also been found to target NEDD9 protein and is downregulated in PC patients (Xia et al., 2018). It is evident that miRNAs display multi-dimensional, multi-angle, and multi-pathway involvement in the regulation of the onset and progression of PC. These examples underscore the critical role of miRNAs in modulating complex molecular mechanisms and highlight their potential as therapeutic targets and biomarkers in PC.

## 3 The role of miRNAs in the diagnosis

The abysmal prognosis of PC is primarily due to its inability to be diagnosed early, which delays early treatment. To date, serum carbohydrate antigen 19–9 (CA19-9) is the only biomarker approved by the Food and Drug Administration (FDA) for PC. However, CA19-9 has poor specificity and sensitivity and is prone to false positives (Luo et al., 2021), making it difficult to use for the early diagnosis of PC. Therefore, there is an urgent need to discover new biomarkers. The stability of mature miRNAs in body fluids, non-invasive screening, and specificity for different cancers have made them increasingly popular for research as PC biomarkers (Xia et al., 2021).

### 3.1 Tissue

The diagnosis of suspicious PC typically requires a biopsy of the primary or metastatic focus. However, the limited amount of tissue obtained during biopsy may result in a misdiagnosis, and distinguishing PC from inflammation can also present difficulties. On the contrary, miRNA analysis has shown promise for identifying PC, even in cases where only a tiny amount of tissue is available. Schultz et al. (2012) found that PC tissue had differential expression of miRNAs compared to normal pancreatic tissue. Out of a total of 664 miRNAs analyzed, 43 were found to be upregulated, while 41 were downregulated in PC tissue. In comparison to chronic pancreatitis (CP), a total of 17 miRNAs exhibited upregulation, while 15 miRNAs showed downregulation in PC tissue. Furthermore, a set of 19 miRNAs was tested for the purpose of distinguishing between PC, ampullary adenocarcinoma, CP, and normal pancreatic tissue; the final research results indicated that these miRNAs had a sensitivity of 98.5%, an accuracy of 97%, and a positive predictive value of 97.8% for diagnosing PC. Certain miRNAs show differential regulation, either upregulation or downregulation, in both PC and normal or inflammatory tissue, which may be helpful for the diagnosis of PC. In recent years, Zhang et al. (2019) demonstrated that the expression levels of miR-25-3p in PDAC are significantly elevated compared to those in non-tumor tissue; experimental evidence further indicates that the upregulation of miR-25-3p stimulates the proliferation and metastasis of PDAC cells in mice, both *in vitro* and *in vivo*. MiR-4282 has been observed to inhibit the migration ability of PC cells (negatively targeting *ABCB5*), indicating that it may also become a promising biomarker for PC (Li and Hou, 2020). However, further studies are needed to establish the clinical relevance of these miRNAs and their interactions with other molecular pathways involved in PC. The translation of these findings from bench to bedside requires comprehensive preclinical and clinical evaluations.

### 3.2 Blood

The detection of miRNAs from blood has higher sensitivity, stability, and accessibility in comparison to tissue detection, giving hope as a novel screening approach for PC. The Schultz team later conducted a case-control study (Schultz et al., 2014), demonstrating that some features of miRNAs in whole blood could be utilized to diagnose PC to a certain extent. They identified two combinations for diagnosis: panel I (miR-145, miR-150, miR-223, miR-636) with a sensitivity of 85% and a specificity of 64%, and panel II (miR-636, miR-26b, miR-223, miR-122, miR-150, miR-145, miR-505, miR-34a, miR-885-5p, miR-126) with all parameters (sensitivity and specificity) at 85%. The study offers promising data on the diagnostic potential of blood-based miRNA panels. Deng et al. (Deng et al., 2016) adopted reverse transcription quantitative PCR (RT-qPCR) as a method to determine the level of miR-25 expression in a total of 303 serum samples. MiR-25 has significant advantages in the differential diagnosis between PC patients and normal individuals compared to CA19-9 and carcinoembryonic antigen (CEA). Furthermore, previous studies (Guz et al., 2021; Xu et al., 2021; Li et al., 2013) have observed that miR-210-3p and miR-1290 may also serve as potential biomarkers for diagnosing PC through serum detection due to their abnormal expression. In a prospective study (Duell et al., 2017), it was discovered that miR-21-5p and miR-30c in plasma showed a significant association with PC 2 or 5 years before patients were diagnosed with this malignant disease, indicating that the utilization of these specific miRNAs derived from plasma holds promise for the early screening of PC in individuals at high risk.

A miRNA regulates various target genes, and a target gene is controlled by multiple miRNAs, forming a complex regulatory network. There are abnormal expressions in the process of the pathogenesis and development of many diseases, including cancers, immune system diseases, etc. Alone as a PC marker obviously lacks specificity; the combination of miRNA and other biomarkers in the blood may be more helpful for detecting PC. Yu et al. (2020) demonstrated that the combined application of CA19-9 and miR-25 showed a higher sensitivity for the early identification of PC compared to using CA19-9 alone or a combination of CA19-9 and CA125, and the area under the curve (AUC) reached 0.985. The conclusion was similarly confirmed by Gong et al. (Gong et al., 2023) through a retrospective research study. These studies underscore the potential for multi-biomarker approaches to improve early detection rates. And the prospective validation studies are essential to confirm these findings in clinical practice. In a study conducted by researchers (Zou et al., 2019), a combination of six specific types of microRNAs (let-7b-5p, miR-192-5p, miR-19a-3p, miR-19b-3p, miR-223-3p, miR-25-3p) in serum was utilized for the purpose of diagnosing PC; the result showed a considerably enhanced diagnostic performance compared to that of a single microRNA. The clinical utility of such panels depends on their reproducibility in larger, independent cohorts and their ability to differentiate PC from other pancreatic diseases accurately. Furthermore, a number of pertinent and significant research endeavors have been conducted, the outcomes of which have been succinctly compiled and presented in Table 1.

**TABLE 1:** Blood biomarkers to identify PC patients and normal controls^a^.

Biomarker	Sample	Sensitivity (%)	Specificity (%)	AUC	Ref.
Panel (miR-636, −26b, −223, −122, −150, −145, −505, −34a, −885-5p, −126)	Whole blood	85.0	85.0	0.930	Schultz et al. (2014)
miR-25+CA19-9	Serum	97.5	90.1	0.985	Yu et al. (2020)
miR-25+CA19-9	Serum	93.3	88.5	0.950	Gong et al. (2023)
miR-18a	Plasma	92.0	94.0	0.937	Morimura et al. (2011)
Panel (miR-16, −196a) +CA19-9	Plasma	92.0	95.6	0.979	Liu et al. (2012a)
Panel (miR-20a, −21, −24, −25, −99a, −185, −191)	Serum	94.0	95.0	0.990	Liu et al. (2012b)
miR-1290	Serum	88.0	84.0	0.960	Li et al. (2013)
miR-125a, −4294, −4476, −4530, −6075, −6799, −6836, −6880	Serum	80.3	97.6	0.953	Kojima et al. (2015)
panel (miR-16, -18a, −20a, −24, −25, −27a, −29c, −30a-5p, −191, -323-3p, −345, −483-5p)	Serum	85.0	90.0	0.950	Johansen et al. (2016)
miR-22-3p	Plasma	97.1	93.3	0.943	Hussein et al. (2017)
miR-642b-3p/miR-885-5p	Plasma	100.0	100.0	1.000	Hussein et al. (2017)
miR-373	Serum	81.0	84.0	0.852	Hua et al. (2017)
miR-133a	Serum	90.6	87.2	0.893	Wang (2020)
miR-122-5p	Serum	98.0	96.0	0.988	Khan et al. (2021)

^a^ The table summarizes the relevant results of experiments with sensitivity and specificity values higher than 80% and AUC greater than 0.85 for individual miRNAs or combinations; sensitivity and specificity values are accurate to 1 decimal place, AUC to 3 decimal places; Ref, reference.

### 3.3 Saliva

Saliva originates from the blood and has been noticed to include multiple molecules that exhibit similarities to those present in the systemic circulation (Miller et al., 2010). The salivary gland has a high blood flow rate and has functional similarities with the pancreas (Tiffo, 2020). These biological characteristics establish a solid foundation for considering salivary miRNA as a potential biomarker for PC. Humeau et al. (2015) conducted RNA extraction from saliva samples and subsequently employed RT-qPCR to screen a total of 94 miRNAs; then proceeded to analyze the expression levels of these miRNAs in saliva samples obtained from PC patients, pancreatitis, pancreatic intraductal papillary mucinous neoplasms, and healthy individuals. It was shown that the expression levels of miR-21, miR-23a, and miR-23b were notably increased in the salivary samples of PC patients; among these microRNAs studied, miR-23 (a, b) has been observed to be upregulated in individuals with precursor lesions of PC, hinting that it may predict the occurrence of PC. According to a report (Xie et al., 2015), compared with patients with benign pancreatic tumors or normal controls, miR-3679-5p in the saliva of PC patients showed notable downregulation after stimulation of the salivary glands, while miR-940 showed upregulation; the combined detection of the two miRNAs achieved a sensitivity of 72% and a specificity of 70% for identifying resectable PC. Koopaie et al. (2022) conducted a systematic evaluation and meta-analysis involving 2,731 subjects (1,465 patients with PC and 1,266 healthy individuals) to evaluate the diagnostic value of salivary non-coding RNA biomarkers in PC, and the results revealed a sensitivity of 82.9% and a specificity of 78.3%, in which miR-1246 and miR-92a displayed reasonable diagnostic accuracy. Moreover, the study showed that stimulated saliva showed higher diagnostic accuracy compared to unstimulated saliva. So far, there remains insufficient research relating to the early screening of PC by detecting salivary miRNAs; however, existing studies indicate that this approach has promise as a potential biomarker.

### 3.4 Urine

Urine originates from blood, the body fluid produced by glomerular filtration and tubular reabsorption, and is frequently employed as a diagnostic tool for identifying various disorders. Debernardi et al. (2015) analyzed urine samples obtained from 46 PC patients, 29 CP patients, and 26 healthy people by RT-qPCR technology. The results demonstrated that PC patients can be distinguished from CP patients due to upregulated miR-223 and miR-204 detected in urine; besides, miR-143, miR-223, and miR-30e were overexpressed in patients with stage I PC compared with the other two groups, among which, the AUC of miR-143 for the diagnosis was 0.862, with sensitivity and specificity of 83.3% and 88.5%, respectively; the specificity of the combination of miR-143 and miR-30e for the diagnosis of PC at this stage reached 96.2%. Compared with stage II-IV PC patients, miR-143, miR-223, and miR-204 were expressed at higher levels in patients with stage I PC. It is obvious that miR-143 and miR-223 could not only identify PC but also have more advantages for PC diagnosed at an earlier stage. However, the relatively small sample size limits the generalizability of these findings. Another study (Ishige et al., 2020) reported that comparing serum and urine samples from PC patients and healthy controls, miR-1246 was highly expressed in PC patients and positively correlated between the two samples, with an AUC of 0.90 for urinary miR-1246, and sensitivity and specificity of 90.2% and 83.3%, respectively, although the study demonstrated no difference in miR-1246 expression in the saliva of PC patients and healthy people. The findings highlight the robustness of miR-1246 as a non-invasive biomarker for PC. The strong correlation between serum and urine levels of miR-1246 suggests its potential for integrated biomarker strategies. It is worth mentioning that Yoshizawa et al. (2020) reported that the ratio of miR-3940-5p to miR-8069 in urine exosomes might serve as a helpful tool for the diagnosis of PDAC. The previous findings indicate that urine miRNA testing, which is both non-invasive and convenient, offers substantial promise as a method of early detection for PC. Furthermore, it may have the potential to differentiate between individuals in the early and advanced stages of this disease.

### 3.5 Stool

Research has also been carried out on the examination of fecal miRNA as a potential biomarker for the diagnosis of PC. Link et al. (2012) analyzed 45 fecal specimens (15 each from PC, CP patients, and healthy controls); the results showed that the expression of miR-196a, miR-216a, miR-143, and miR-155 from PC patients was relatively low, which was in contrast to the later finding of Yang et al., who found high expression of miR-155 in PC (Yang et al., 2014). In addition, Yang’s team (Yang et al., 2014) experimentally demonstrated that fecal miR-21 expression levels in patients with this malignant disease were higher than those in normal people, while miR-216 expression levels were lower. In a separate study (Ren et al., 2012) the levels of miR-181b and miR-210 expression in fecal samples from the PC group were significantly higher compared to those in the healthy control group (AUC of miR-181b-0.745; AUC of miR-210–0.772). According to existing studies, fecal miRNAs have the potential to become diagnostic markers for PC; however, the limited sample size and partially conflicting results necessitate further research.

### 3.6 Pancreatic juice

Pancreatic juice (PJ) functions as a valuable source of potential biomarkers, such as proteins and miRNAs, which are closely linked to the processes of tumorigenesis and development. These molecules in PJ have the potential to facilitate the early detection of pancreatic lesions through the application of genomes, transcriptomics, or other associated technologies. Sadakari et al. (2010) identified the presence of miRNAs in PJ and reported for the first time that high expression of miR-21 and miR-155 in PJ might serve as a promising diagnostic biomarker for PDAC. It is important to note that, subsequent to the extraction of pancreatic exosomes by Nakamura et al. (2019), it was detected that the expression of miR-21 and miR-155 in pancreatic exosomes from PC patients was significantly higher than that in CP patients; the sensitivity, specificity, and accuracy of the joint of these two miRNAs in diagnosing PC were 96%, 75%, and 91%, respectively, which was more advantageous than pancreatic fluid cytology diagnosis. This approach shows promise, particularly in improving sensitivity and overall diagnostic performance. However, the specificity remains moderate, indicating a need for additional markers or methods to reduce false positives and further refine the diagnostic criteria. The results of a study (Wang J. et al., 2014) suggested that elevated levels of miR-205, miR-210, miR-492, and miR-1247 in PJ collectively diagnose PDAC with a sensitivity of 87% and specificity of 88%; when these miRNAs combined with CA19-9, the sensitivity increased to 91% and specificity to 100%. These specific microRNAs were also associated with decreased overall survival (OS) in PDAC patients; furthermore, elevated levels of miR-205 and miR-210 were related to lymph node metastasis (LNM), while high expression of miR-205 was also associated with poorly differentiated tumors, both of which are indicators of an unfavorable prognosis. The concentration of miRNAs in PJ is higher than that in serum or plasma, and its presence may be sustained over an extended period, making it a promising biomarker for the early diagnosis of PC. Nonetheless, the extraction of PJ is an invasive procedure often collected through endoscopic retrograde cholangiopancreatography (ERCP) in clinical practice, which may potentially limit its clinical application. The current studies on miRNAs in body fluids other than blood are detailed in Table 2.

**TABLE 2:** Other body fluid biomarkers to identify PC patients and non-PC controls^a^.

Sample	MicroRNA	Expression	Sensitivity	Specificity	AUC	Ref.
Saliva	miR-21	UR	71.0%	100.0%	NA	Humeau et al. (2015)
	miR-23a	UR	86.0%	100.0%	NA	Humeau et al. (2015)
	miR-23b	UR	86.0%	100.0%	NA	Humeau et al. (2015)
	miR-3679-5p + -940	NA	72.5%	70.0%	NA	Xie et al. (2015)
	miR-4644	UR	75.0%	76.9%	0.763	Machida et al. (2016)
	miR-1246+-4644	NA	83.3%	92.3%	0.833	Machida et al. (2016)
Urine	miR-143	UR	83.3%	88.5%	0.862	Debernardi et al. (2015)
	miR-223	UR	83.3%	76.9%	0.795	Debernardi et al. (2015)
	miR-30e	UR	83.3%	80.8%	0.853	Debernardi et al. (2015)
	miR-143+-30e	NA	83.3%	96.2%	0.923	Debernardi et al. (2015)
	miR-223+-30e	NA	83.3%	92.3%	0.891	Debernardi et al. (2015)
	miR-1246	UR	90.2%	83.3%	0.900	Ishige et al. (2020)
Stool	miR-196a, −216a, −143, −155	DR^b^	NA	NA	NA	Link et al. (2012)
	miR-155	UR^b^	76.7%	73.3%	0.719	Yang et al. (2014)
	miR-21+-155+-216	NA	83.3%	83.3%	0.867	Yang et al. (2014)
	miR-181b	UR	84.6%	84.6%	0.745	Ren et al. (2012)
Pancreatic juice	miR-21, -155	UR	NA	NA	NA	Sadakari et al. (2010)
	miR-210	UR	76.0%	95.0%	0.840	Wang J. et al. (2014)
	miR-492	UR	73.0%	82.0%	0.800	Wang J. et al. (2014)
	miR-205+-210+-492+-1,247	NA	87.0%	88.0%	0.920	Wang J. et al. (2014)
	miR-205+-210+-492+-1,247+CA19-9	NA	91.0%	100.0%	0.990	Wang J. et al. (2014)

^a^ The table summarizes the relevant experiments of body fluid miRNAs as biomarkers other than blood and has excluded the results with sensitivity and specificity lower than 70% and AUC, lower than 0.7; sensitivity and specificity values are accurate to 1 decimal place, AUC, to 3 decimal places; NA, not available; UR, upregulation; DR, downregulation; Ref, reference.

^b^ The two experiments involved opposite results of miR-155 expression, and the conclusions need to be further researched.

Numerous studies have confirmed that miRNAs are small molecules with highly potential for diagnosing PC. They are often stable, easy to collect, and safe, and their combination with other biomarkers may have brighter prospects. Nevertheless, their clinical application is still in its infancy. The low sensitivity or high cost of measurement methods, as well as the complex workflow, have limited the use of miRNA as a clinical cancer biomarker. An American team (Yan et al., 2023) reported a one-pot isothermal Cas12-based assay (EXTRA-CRISPR), which was used to quantify miR-21, miR-196a, miR-451a, and miR-1246 in extracellular vesicles (EVs), based on liquid biopsies for the diagnosis of PDAC, and parallel RT-qPCR analysis of the same clinical samples verified the validity of the use of EXTRA-CRISPR for diagnosis. Improving the method to detect miRNAs would undoubtedly help to promote the clinical use of this crucial regulatory factor and enhance the efficiency and precision of PC diagnosis.

## 4 The role of miRNAs in the treatment

The close connection between miRNAs and the various biological processes involved in the onset, progression, infiltration, and spread of PC suggests that miRNAs could become novel therapeutic targets for managing this disease. The exploration of miRNA-related targeted therapy strategies is ongoing and aims to improve the poor prognosis associated with PC.

### 4.1 As regulatory factors in traditional treatments

Currently, a minority of PC patients can undergo surgery after a definite diagnosis, but most still rely on chemotherapy, radiotherapy, or immunotherapy as the main treatment methods. Gemcitabine (GEM) is the fundamental drug for chemotherapy in patients at all stages of PC, but its overall effectiveness frequently falls short of expectations. The limited efficacy of this treatment regimen might be attributed to drug resistance (Koltai et al., 2022), which may be related to the TME, epigenetics, and vascular systems. However, the exact mechanism remains uncertain. Gu et al. (2020) identified differential expression of miR-485-3p, miR-574-5p, miR-584-5p, and miR-3178 in GEM-resistant PC cells. Through tumor immune infiltration analysis, they found that the activation of the miRNA-mRNA regulatory network in drug-resistant PC cells may affect CD4 (+) memory T cells in the tumor immune microenvironment, which may impact the survival and prognosis of patients. Subsequently, the research team (Gu et al., 2022) discovered that miR-3178 accomplishes expression upregulation through the ABC transporter proteins, which are mediated by the RhoB/PI3K/Akt signaling pathway, thereby promoting drug resistance in GEM, indicating that miR-3178 can be considered a novel target for enhancing chemotherapy sensitivity to treat PC. Liu et al. (2021) revealed that miR-3662 plays a regulatory role in the metabolism of PC cells by engaging in a negative feedback loop of HIF-1α, sequentially reversing GEM drug resistance, which shows that the combined application of miR-3662 and GEM can be a potential therapeutic strategy for PC patients. The resistance and susceptibility of PC cells to other chemotherapy agents can also be regulated by miRNAs. According to a study (Ouyang et al., 2021), miR-499a-5p was observed to be overexpressed in PC cells that exhibited resistance to 5-Fluorouracil (5-FU), which contributes to both 5-FU resistance and enhanced cell proliferation by targeting *PTEN* and then activating the PI3K/Akt pathway; conversely, the inhibition of miR-499a-5p expression was shown to promote apoptosis in PC cells, inhibit cell proliferation and migration, as well as improve the effectiveness of 5-FU chemotherapy. Lee et al. (2023) found that blocking the expression of miR-1976 enhances the chemosensitivity of PC cells in a dependent manner by promoting the apoptosis gene *XAF1*; based on the results, they proposed that developing a miR-1976 inhibitor combined with chemotherapeutic drugs can enhance the chemotherapy effect and anti-tumor effect. In addition, it has been demonstrated that miR-20a-5p, miR-21, and miR-181a-5p show associations not only with chemotherapy sensitivity but also with the ability to predict the post-chemotherapy response of patients with PC to GEM, 5-FU, and FOLFIRINOX (a combination of three chemotherapy drugs), respectively; the overexpression of these miRNAs frequently signifies chemotherapy failure (Lu et al., 2019; Donahue et al., 2014; Meijer et al., 2020). Thus, miRNAs can be used as guiding factors in the treatment process, providing early guidance to clinicians for adjusting chemotherapy regimens and selecting the most optimal strategy, ultimately leading to enhanced chemotherapy efficacy and improved patient survival rates.

Moreover, researchers have found that miRNAs also play an important role in the radiotherapy process of PC patients. Zhang et al. (2015) successfully cultured a PC cell line with radiotherapy resistance and observed a significant downregulation in the expression of miR-216a; Further research proved that the overexpression of miR-216a directly interacted with the 3′-UTR of *beclin-1* (an autophagy gene), inhibiting PC cell growth and promoting apoptosis of cells with radiotherapy resistance. The ability of miR-216a to modulate autophagy and apoptosis provides a promising strategy for enhancing the effectiveness of radiotherapy. However, translating these findings into clinical practice requires further validation in animal models and human trials. McGrath et al. (2022) utilized experiments and bioinformatics tools to prove that the regulation of miR-31 can induce changes in the expression of GPx8, thereby enhancing the susceptibility of PC cells to radiation. Similarly, upregulating the expression of miR-216b, miR-23b, miR-374, or inhibiting the expression of miR-620 and miR-99b has been shown to serve as a tumor radiation sensitizer, with the hope of assisting in the clinical treatment of PC (Egeli et al., 2016; Wang et al., 2013; Baek et al., 2016; Huang et al., 2015; Wei et al., 2013). In contrast to radiotherapy and chemotherapy, which employ external approaches to kill cancer cells, immunotherapy harnesses the inherent capabilities of the body’s immune system to target and combat cancer cells. Xi et al. (2020) confirmed that miR-340 is a crucial regulator of anti-tumor immunity, which increases macrophage-mediated phagocytosis by downregulating CD47 on PC cells, inhibits the proliferation and migration of cancer cells, promotes apoptosis, and strengthens anti-tumor effects. Additionally, a study (Jia et al., 2017) indicated that PD-L1 is regulated by miR-142-5p, whose overexpression enhances anti-tumor immunity by blocking the PD-L1/PD-1 pathway.

In summary, miRNAs have a substantial regulatory function in traditional anti-tumor therapies, including radiation, chemotherapy, and immunotherapy. Proper utilization of oncomiRs and tsmiRs, such as by the overexpression of tsmiRs or the inhibition of oncomiRs, has the potential to achieve sensitization during chemoradiotherapy, activate or enhance the autoimmune system, improve treatment efficacy, and prolong survival in PC patients. Despite these promising findings, the clinical application of miRNA-based therapies faces challenges related to safety, delivery, and specificity. Extensive preclinical and clinical studies are required to validate these approaches and develop robust delivery systems. A summary of the role of miRNAs in routine treatments for PC is presented in Table 3.

**TABLE 3:** The role of miRNAs in conventional therapies^a^.

MiRNA	Expression	Treatment	Ref.
miR-3178	UR	Promote resistance (GEM)	Gu et al. (2022)
miR-3662	DR	Reverse resistance (GEM)	Liu et al. (2021)
miR-499a-5p	UR	Promote resistance (5-FU), cell proliferation	Ouyang et al. (2021)
miR-1976	DR	Enhance chemotherapy sensitivity	Lee et al. (2023)
miR-216a, −31, −216b, −23b, −374, −620, −99b	UR	Enhance radiotherapy sensitivity	Zhang et al. (2015), McGrath et al. (2022), Egeli et al. (2016), Wang et al. (2013), Baek et al. (2016), Huang et al. (2015), Wei et al. (2013)
miR-340, −142-5p	UR	Enhance anti-tumor immunity	Xi et al. (2020), Jia et al. (2017)

^a^ UR, upregulation; DR, downregulation; Ref, reference.

### 4.2 MiRNA-based gene therapy

#### 4.2.1 MiRNA vectors for PC

Gene therapy has enormous potential in the realm of cancer treatment. As essential regulatory factors in the cancer process, miRNAs have limited exogenous delivery due to negative charge repulsion and the hydrophilicity of cell membranes. If miRNAs are to be applied for gene therapy, effective carriers urgently need to be delivered through the dense fibrous matrix, enhancing their effective concentration in tumor tissue and exerting anti-tumor effects (Kurtanich et al., 2019). Viral vectors after detoxification are commonly employed to deliver non-coding RNAs to cancer cells, including adenoviruses (ADs), adeno-associated viruses (AAVs), and retroviruses (RVs) (Galanopoulos et al., 2021). A study (Chen et al., 2013) reported the feasibility of utilizing a sensor named “Asensor” with AAV as a carrier, then demonstrated its potential for real-time monitoring of miRNA function in PC living cells, which provides a precise and convenient method for basic research on miRNAs. Furthermore, Chaudhary et al. (Chaudhary et al., 2017) observed that the overexpression of miR-205 through the utilization of lentivirus as a vector blocks the proliferation of PC cells and strengthens the chemical sensitivity of PC stem cells, surpassing the effectiveness of adenovirus as a vector. Significantly, this method does not impact the production of endogenous miRNAs but might result in adverse reactions such as the activation of oncogenes and genetic toxicity.

The toxicity and immunogenicity of viruses limit their practical clinical applications; the use of nanoparticles as carriers in gene therapy is regarded as a safer option. Overexpression of the tumor suppressor factor miR-145 can lead to downregulation of HER-2, MUC13, and pAKT, thereby inhibiting the proliferation, migration, and invasion of PC cells (Setua et al., 2017). The nanoparticle complex based on this factor (miR-145-MNPF) has been proven to effectively restore the function of miR-145 in PC cells, attaining anti-tumor purposes (Setua et al., 2017). Li et al. (2017) developed a targeted therapeutic strategy combining miR-21 antisense oligonucleotides (ASO-miR-21) and GEM using nanoparticles as carriers, which synergistically inhibit the proliferation of EMT and PC cells by upregulating the tumor suppressor targets *PDCD4* and *PTEN*; whether compared with ASO-miR-21 or GEM alone, the anticancer effect of this combinational regimen is better. It is worth mentioning that a discovery made by Karimnia et al. (2023) shows that the utilization of photodynamic stromal depletion (PSD, a photodynamic therapy that utilizes visible or near-infrared light to destroy connective tissue) has the ability to upregulate the expression of PDCD4, further improving the delivery of therapeutic miRNA nanomedicine and augmenting its anti-tumor efficacy. Moreover, miRNA-based assembled nanoparticles have been shown to transfect relevant macrophages in PC tissue and further influence immunotherapy (Parayath et al., 2021). Targeting macrophages with miRNA-based nanoparticles represents a promising strategy to modulate the tumor immune microenvironment.

In addition to viruses and nanoparticles, exosomes possess inherent stability and have the ability to evade macrophage phagocytosis and lysosomal degradation. Presently, several studies also exploit exosomes as vectors for treating PC. Zuo et al. (2020) conducted a study wherein they isolated exosomes from cells and subsequently applied ultrasonic techniques to synthesize miR-34a coated with exosomes (exomiR-34a), which were capable of penetrating the cell membrane and significantly inhibiting the development of PC *in vitro* and *in vivo*. It has also been reported that exosomes offer the capability to transport exogenous miR-145-5p to PC cells, resulting in the suppression of malignant proliferation and the stimulation of cell cycle arrest (Ding et al., 2019). Exosomes provide a natural and efficient delivery system for miRNAs, with advantages in stability and biocompatibility. However, the scalability of exosome production and the standardization of isolation and loading techniques are critical challenges that must be addressed to facilitate their clinical application.

In general, the application of viruses, nanoparticles, and exosomes as vectors holds promise as viable options for the delivery of miRNA-related gene medicine and targeted therapy *in vivo*. However, each method comes with its unique challenges and limitations that need to be addressed through rigorous research and clinical validation.

#### 4.2.2 Participation in oncolytic virus therapy

Oncolytic virus (OVs) therapy is a novel type of targeted immunotherapy. OVs are specifically expressed locally through armed therapeutic transgenes, stimulating the body to produce anti-tumor immune responses, or selectively replicating using unique signaling pathways in cancer cells to selectively eliminate tumor cells through lysis, resulting in anti-tumor immunity.

The efficacy, specificity, and safety of OV therapy for PC are further improved if miRNAs are engineered to be inserted, which has been confirmed by relevant research reports in recent years (Bommareddy et al., 2018). Raimondi et al. (2021) designed an oncolytic adenovirus (AdNuPARmE1A-miR222-S) that binds to the tumor suppressor factor miR-222 at the locus, which is highly active *in vivo* and enhances the cytotoxic effect of the adenovirus, effectively controlling the progression of PC. MiR-99b and miR-485 were also shown to act as sensitizers for oncolytic adenovirus therapy of PC following *in vitro* and *in vivo* experiments (Rovira-Rigau et al., 2019). Additionally, a responsive oxygen-self-supplying adv-miRT-CAT-KR (adv-MCK) cascade reaction system was constructed to improve hypoxia in PC (Chen et al., 2024). This system, utilizing an oncolytic adenovirus targeted by miRNAs, selectively replicates in PC cells and generates endogenous oxygen to enhance the oncolytic effect, specifically improving hypoxia and activating antitumor immunity. Furthermore, Singh et al. (2021) designed and validated a 5-FU-based chemo-viral therapy with oncolytic measles virus, which enhances tumor specificity and strengthens toxicity to PC cells through miR-148a regulation of vector tropism; the substantial efficacy in reducing tumor size and prolonging progression-free survival was also verified. These studies demonstrated promising results with miRNA-engineered OVs, the long-term safety and potential immune responses against the viral vector need further investigation. The complexity of combining multiple therapeutic modalities also necessitates detailed studies on drug-virus interactions, dosing strategies, and patient selection criteria to maximize therapeutic benefits and minimize adverse effects. Liver damage may occur with OV therapy. An *in vitro* and *in vivo* experiment by Bofill-De Ros et al. (2014) confirmed that the use of oncolytic adenoviruses controlled by miR-148a and miR-216a with high regulatory ability can reduce normal pancreatic and liver tissue damage without interfering with miRNA activity, which has stronger safety and anti-PC efficacy. This research is of great importance in enhancing the safety of OVs.

At present, the involvement of miRNAs in oncolytic viral therapy is a potentially advantageous strategy for treating cancer. Still, the specific impact of the widely dysregulated miRNA spectrum on virus replication remains uncertain. In order to improve the oncolytic efficacy of viruses, it is imperative to undertake further research on miRNAs with specific expression as well as to reestablish crucial cellular gene levels related to productive viral infection. Challenges related to long-term safety, immune responses, and clinical scalability remain. Further research is essential to optimize miRNA-engineered OVs, addressing issues such as off-target effects and delivery efficiency, with the ultimate goal of translating these protocols into clinical applications.

#### 4.2.3 MiRNA mimics and inhibitors

Depending on the function of oncomiR and tsmiR, miRNA mimics (chemically modified double-stranded RNAs *in vitro*) and miRNA inhibitors (single-stranded RNAs) currently exist as two specific modalities of miRNA-based drug therapy. The former uses miRNA mimics to supplement tsmiRs, simulating the function of corresponding miRNAs naturally produced in the body; the latter utilizes synthesized miRNA inhibitors to form complementary sequences with targeted oncomiRs, inhibiting the function of corresponding miRNAs.

MRX34 is a synthetic mimic of miR-34a (tsmiR), the first miRNA mimic to undergo evaluation in clinical trials. Beg et al. (2017) evaluated the clinical activity, safety, and pharmacokinetics of the drug; they administered MRX34 intravenously twice weekly to adult patients with solid tumors (including PC) for 4 weeks. The results indicated that the evaluated compound exhibited anti-cancer properties against advanced solid tumors; however, the study was terminated due to the occurrence of significant adverse effects associated with the immune system (Hong et al., 2020). While these results demonstrate the potential of MRX34, the severe immune-related adverse effects highlight a significant limitation that needs to be addressed in future studies. Relevant preclinical trials are also ongoing. Ghosh et al. (2023) injected TTX-MC138 (miR-10b inhibitor conjugated to ultrasmall iron oxide nanoparticles) intravenously weekly into mice carrying orthotopic xenografts derived from human PC cells. They found that compared with the control group, the tumor growth rate of mice treated with TTX-MC138 as a single drug was significantly reduced and 40% of the tumors were completely relieved; no tumor progression or metastasis was found in the anatomy 10 weeks after the treatment was stopped, which proved the potential of TTX-MC138 in PC treatment. Up to now, the non-clinical studies and Phase 0 clinical trials of this drug are underway (Medarova, et al., 2024). In non-human primates, the organs with the highest drug uptake were the liver, heart, lung and spleen, with an average whole blood half-life of 12.2 ± 2.3 h (mean ± standard deviation). Preliminary clinical data from Phase 0 trials (NCT05908773) indicated good tolerability, long circulation half-life, and drug accumulation in metastatic lesions. Enrollment, data monitoring, and analysis is ongoing. MiRNA mimics and inhibitors often require vector delivery to function. Recently, Yuen et al. (2022) developed a new approach for treating PC utilizing miRNA, which replaced the uracil bases with 5-FU in the guidance chain of tsmiR; they demonstrated that this 5-FU modified miRNA mimic has higher efficacy in inhibiting cancer cell proliferation and does not require any carrier, suitable for various cancers like PC. Similar approaches may become a platform technology for future nucleic acid-based therapies.

A single miRNA has the capability to simultaneously regulate numerous genes and signaling pathways, subsequently exerting an influence on a wide range of biological functions. Therefore, the focused regulation of a specific miRNA can provide enormous effects. Numerous *in vitro* and *in vivo* experiments have demonstrated the great potential of cancer-therapeutic miRNAs (Gurbuz and Ozpolat, 2019). MiRNA mimics and inhibitors present a promising avenue for the development of targeted cancer therapies. Nevertheless, it is essential to take into account the potential medication toxicity of miRNA in normal cells, as miRNA can be enriched in both tumor and normal cells. MiRNAs act on hundreds or thousands of genes, making it challenging to achieve specific gene regulation, which can result in unpredictable side effects. In the future, only by addressing the issue of specificity might miRNAs be truly applicable in clinical settings.

## 5 The role of miRNAs in prognosis

Multiple miRNAs associated with tumor progression and invasiveness can serve as potential prognostic indicators for several diseases. The potential prognostic value of miR-21 in predicting survival among patients with PC has been extensively studied. Individuals with overexpression of miR-21 tend to have a much inferior survival outcome compared to those with no detectable expression of miR-21 in lymph node-negative PC tissue (median 27.7 months versus 15.2 months) (Dillhoff et al., 2008). Ali et al. (2022) conducted an international multicenter study involving 686 patients, which demonstrated a significant correlation between high expression of miR-21, tumor size, and lymph node metastasis, as well as a considerable decrease in OS, indicating poor prognosis. However, these patients may benefit more from adjuvant chemotherapy containing GEM. These results predominantly reflect a correlation between miR-21 levels and survival outcomes. Also, a study (Karasek et al., 2018) reported that the expression level of miR-21 in preoperative plasma acts as an independent prognostic factor for patients undergoing PC surgery. These results highlight strong evidence linking high miR-21 expression to poor prognosis in PC patients. However, further validation and standardization are needed in detection methods, patient cohort characteristics, and clinical application to fully establish miR-21 as a reliable prognostic biomarker. Similarly, overexpression of miR-10b was found to be involved in the invasiveness of PC cells, predicting an unfavorable prognosis in such patients, while low-expression individuals are more likely to achieve surgical resection, a good response to neoadjuvant therapy and delayed tumor metastasis, further increasing the survival rate (Nakata et al., 2011; Preis et al., 2011). Greither et al. (2010) confirmed that elevated expression levels of miR-155, miR-203, miR-210, and miR-222 not only promote the activity of PC cells but also show an extensive association with the potential mortality risk (increased 6.2 times) and a poor prognosis among patients with PC. These findings suggest that patients with low expression of these miRNAs have better surgical and therapeutic outcomes. Indeed, there exist instances in which high expression of various miRNAs is indicative of a beneficial prognosis. A report (Yu et al., 2010) suggested that the five-year survival rate of the miR-200c overexpression group is 33.5%, which is significantly higher than 11.2% of the low-expression group. In addition, a meta-analysis (Zhao et al., 2020) evaluated the value of miRNAs in the prognosis of PC, which revealed that PC patients with high expression of miR-451a and miR-1290 originating from the blood had prominent short OS (*P* < 0.05), and patients with upregulated miR-17-5p, miR-23a, miR-221 or downregulated miR-29c, miR-126, and miR-218 originating from the tissue samples also showed poor prognosis (*P* < 0.05).

In the treatment group using GEM, PC patients with high expression of miR-142-5p and miR-204 had significantly longer survival periods compared to those with low expression; however, the discovery was not applicable in untreated or treated patients without GEM (Ohuchida et al., 2011). In addition, Van der Sijde et al. (2021) collected the serum of PDAC patients before and after treatment applying FOLFIRINOX, compared the serum of patients with and without disease progression during the treatment period, and analyzed the miRNA expression before and after chemotherapy. They ultimately found that before chemotherapy, the expression of miR-373-3p within the serum of patients with later progression was higher than that of patients without progression; after one cycle of treatment with FOLFIRINOX, the serum-derived miR-194-5p expression of patients with disease progression was downregulated. This suggests that the high expression of serum miR-373-3p before chemotherapy and the low expression of miR-194-5p after chemotherapy can predict the early progression of PC during treatment with FOLFIRINOX. In another study, Demiray et al. (2022) demonstrated that among patients receiving FOLFIRINOX, those with low serum levels of let-7c experienced significantly earlier disease progression compared to those with high levels. Specifically, low let-7c levels were associated with a 2.104-fold increased risk of early progression. The study highlights let-7c as a potential prognostic biomarker for patients undergoing FOLFIRINOX therapy.

Recently, enormous research endeavors have been conducted to develop prognostic prediction models for patients with PC based on combinations of miRNAs. These studies have yielded satisfactory outcomes. Nishiwada et al. (2020) identified 6-miRNA markers (miR-92b-3p, miR-155-5p, miR-196b-5p, miR-365a-5p, miR-629-5p, and miR-675-3p) for detecting LNM in PDAC patients (AUC = 0.73); survival analysis was performed to assess the prognostic potential of these markers, revealing promising results. In their research, Wolfe et al. (Wolfe et al., 2020) conducted a risk study based on the differential expression of four microRNAs (miR-29c, miR-155, miR-125a, and miR-200b) in PC surgical specimens. The researchers ultimately found that patients classified in the high-risk group (based on risk score) exhibited a considerably worse two-year survival rate compared to those in the low-risk group (27.7% versus 52.2%), with higher local and distant recurrence rates, indicating an inferior prognosis. Detailed are provided in Table 4.

**TABLE 4:** The role of miRNAs in prognosis^a^.

MiRNA	Sample	Expression	Prognosis	Influence	Ref.
miR-21	Tissue/Plasma	High	Poor	OS, MST, O, LNM, T	Lu et al. (2019), Dillhoff et al. (2008), Ali et al. (2022), Karasek et al. (2018)
miR-10b	Tissue	High	Poor	OS, O, NT, M	Nakata et al. (2011), Preis et al. (2011)
miR-155, -203, −210, −222	Tissue	High	Poor	OS	Greither et al. (2010)
miR-200c	Tissue	High	Good	OS	Yu et al. (2010)
miR-451a, −1290	Blood	High	Poor	OS	Zhao et al. (2020)
miR-17-5p, −23a, −221	Tissue	High	Poor	OS	Zhao et al. (2020)
miR-29c, −126, −218	Tissue	Low	Poor	OS	Zhao et al. (2020)
miR-204, -142-5p	Tissue	Low	Poor	OS(GEM)	Ohuchida et al. (2011)
miR-373-3p, miR-194-5p	Serum	High, Low	Poor	OS(FOLFIRINOX)	Van der Sijde et al. (2021)
let-7c	Serum	Low	Poor	OS(FOLFIRINOX)	Demiray et al. (2022)
miR-20a-5p	Plasma	High	Poor	PFS(GEM)	Donahue et al. (2014)
miR-181a-5p	Plasma	Low	Good	PFS(FOLFIRINOX)	Meijer et al. (2020)

^a^ OS, overall survival; MST, median survival term; O, operability; LNM, lymphatic metastasis; T, tumor size; NT, neoadjuvant therapy; M, metastasis; PFS, progression free survival; Ref, reference.

Overall, the differential expression of miRNAs is closely related to the prognosis of PC patients, specifically predicting the possibility of surgical resection, the effectiveness of routine treatments, and the occurrence of metastasis. Combinatorial detection of miRNAs has emerged as a valuable approach for enhancing prognostic accuracy, highlighting the need for continued research to fully integrate these biomarkers into clinical practice.

## 6 Conclusion

To date, miRNAs are involved in every process of PC, including onset, progression, metastasis, and drug resistance, highlighting their critical role in this highly aggressive disease. Numerous studies have demonstrated that differentially expressed miRNAs are considered potential biomarkers for PC diagnosis and prognosis, especially when combined with other miRNAs or clinical markers such as CA19-9, showing promising potential. However, more large-scale prospective studies are needed to compare the sensitivity, specificity, and accuracy of various diagnostic methods, expand sample sizes, and ultimately establish standardized diagnostic and treatment procedures based on miRNA genomics. On the therapeutic side, the use of viruses, nanoparticles, and exosomes as vectors holds promise for gene-drug delivery and *in vivo* targeted therapies. The design of specific oncolytic viruses based on miRNAs is also a potential candidate to treat PC patients. MiRNA-based medicines offer unique advantages, including easy identification and screening and the ability to modulate multiple disease pathways simultaneously. Nevertheless, the drug development process requires resolving numerous issues, such as optimizing precise targeting processes, mitigating immunogenic responses, and determining appropriate dosage regimens. Currently, miRNA research has entered the clinical stage from the laboratory, with a few preliminary clinical trials in progress. TTX-MC138 is an anticipated product, showing promising initial data. Furthermore, exploring the relationship and mechanism of interaction between miRNAs and traditional treatments for PC patients presents new perspectives for treatment strategies. Further research and verification are required to explore the potential for future clinical applications of related pharmaceutical compounds.

In conclusion, miRNAs hold significant promise as diagnostic and prognostic biomarkers and therapeutic agents in PC. Their integration into clinical practice, however, requires extensive validation through large-scale clinical trials and continuous exploration of their mechanisms and interactions with existing treatments. By addressing these challenges, miRNA-based approaches could revolutionize the management and treatment of PC, offering hope for improved patient outcomes and personalized medicine.
